# The Yield of Genetic Testing and Putative Genetic Factors of Disease Heterogeneity in Long QT Syndrome Patients

**DOI:** 10.3390/ijms252211976

**Published:** 2024-11-07

**Authors:** Anna Bukaeva, Alexandra Ershova, Maria Kharlap, Anna Kiseleva, Vladimir Kutsenko, Evgeniia Sotnikova, Mikhail Divashuk, Maria Pokrovskaya, Elizaveta Garbuzova, Anastasia Blokhina, Oksana Kopylova, Evgenia Zotova, Anna Petukhova, Anastasia Zharikova, Vasily Ramensky, Marija Zaicenoka, Yuri Vyatkin, Alexey Meshkov, Oxana Drapkina

**Affiliations:** 1 National Medical Research Center for Therapy and Preventive Medicine, 101990 Moscow, Russia; alersh@mail.ru (A.E.); kharlapmaria@yahoo.com (M.K.); sanyutabe@gmail.com (A.K.); vlakutsenko@ya.ru (V.K.); divashuk@gmail.com (M.D.); mpokrovskaia@list.ru (M.P.); vostryakova.elizaveta@gmail.com (E.G.); blokhina0310@gmail.com (A.B.); anna.petukhova.96@gmail.com (A.P.); azharikova89@gmail.com (A.Z.); ramensky@gmail.com (V.R.); marija.zaicenoka@gmail.com (M.Z.); vyatkin@gmail.com (Y.V.); meshkov@lipidclinic.ru (A.M.); drapkina@bk.ru (O.D.); 2 All-Russia Research Institute of Agricultural Biotechnology, 127550 Moscow, Russia; 3 Faculty of Bioengineering and Bioinformatics, Lomonosov Moscow State University, 119991 Moscow, Russia; 4 MSU Institute for Artificial Intelligence, Lomonosov Moscow State University, 119991 Moscow, Russia; 5 Moscow Center for Advanced Studies, 123592 Moscow, Russia

**Keywords:** long QT syndrome, genetic diagnosis, genetic testing, whole exome sequencing, Schwartz score, clinical heterogeneity, genetic modifiers

## Abstract

Genetic overdiagnosis of long QT syndrome (LQTS) becomes a critical concern due to the high clinical significance of DNA diagnosis. Current guidelines for LQTS genetic testing recommend a limited scope and strict referral based on the Schwartz score. Nevertheless, LQTS may be underdiagnosed in patients with borderline phenotypes. We aimed to evaluate the total yield of rare variants in cardiac genes in LQTS patients. The cohort of 82 patients with LQTS referral diagnosis underwent phenotyping, Schwartz score counting, and exome sequencing. We assessed known LQTS genes for diagnostics, as per guidelines, and a broader set of genes for research. Diagnostic testing yield reached 75% in index patients; all causal variants were found in *KCNQ1*, *KCNH2*, and *SCN5A* genes. Research testing of 248 heart-related genes achieved a 50% yield of molecular diagnosis in patients with a low Schwartz score (<3.5). In patients with LQTS-causing variants, each additional rare variant in heart-related genes added 0.94 points to the Schwartz score (*p* value = 0.04), reflecting the more severe disease in such patients than in those with causal variants but without additional findings. We conclude that the current LQTS genetic diagnosis framework is highly specific but may lack sensitivity for patients with a Schwartz score <3.5. Improving referral criteria for these patients could enhance DNA diagnosis. Also, our results suggest that additional variants in cardiac genes may affect the severity of the disease in the carriers of LQTS-causing variants, which may aid in identifying new modifier genes.

## 1. Introduction

Over the last decades, inherited cardiac diseases have become one of the most studied groups of monogenic disorders. Being responsible for the majority of sudden cardiac deaths (SCD) in young people [1,2], genetic heart diseases, particularly inherited cardiomyopathies and cardiac channelopathies, remain the objects of special attention in cardiology, and the more we know about their genetic basis, the more awareness and demand for DNA diagnosis arises among both clinicians and patients. Contemporary recommendations for genetic testing in cardiac diseases [3] aim, among other things, to clearly define the goals and scope of genetic testing in order to avoid overtesting and overdiagnosis. In the case of long QT syndrome (LQTS), the genetic diagnosis directly contributes to risk stratification and choice of therapeutic strategy [4,5]. Accordingly, an incorrect or excessive diagnosis in this case is even more dangerous since it may lead to unnecessary lifestyle changes, medications, and surgical interventions [6]. Therefore, extensive genetic testing for LQTS is not encouraged in the current clinical setting [7].

Without disputing this approach, in real clinical practice, we face difficulties that cannot be solved by strict adherence to the guidelines. For instance, patients with a referral diagnosis of LQTS may have a borderline phenotype that is often indistinguishable from a false positive clinical diagnosis [8]. Another problem is that, despite the high clinical actionability of genetic testing for LQTS [9], the correct assessment of SCD risk may still not be straightforward [10] because of the clinical variability between the carriers of the same variants, even within one pedigree. Unraveling this variability is still challenging; the common opinion on the question is that there are distinct genetic modifiers affecting the phenotype in different patients [11,12]. As known, genetic modifiers may be either common single nucleotide variants (SNPs) with small individual effect sizes or rarer SNPs with intermediate effect sizes between ultra-rare Mendelian variants and common modifiers [13]. The effect of common SNPs on QTc interval in the general population has been investigated in a number of genome-wide association studies (GWAS) [14,15], but the polygenic risk score developed to estimate the possible impact of such variants on LQTS patients brought no clinically valuable results [16,17]. The rare variants of intermediate non-Mendelian effects and their possible impact on LQTS patients have not been systematically studied. So, in the present study, we aimed to evaluate the total yield of genetic findings in a wide spectrum of cardiac genes among the patients referred to the Expert Arrhythmology Center as having LQTS and to find whether the possible burden of rare SNPs affects the phenotypic severity of the disease.

## 2. Results

**Figure 1:**
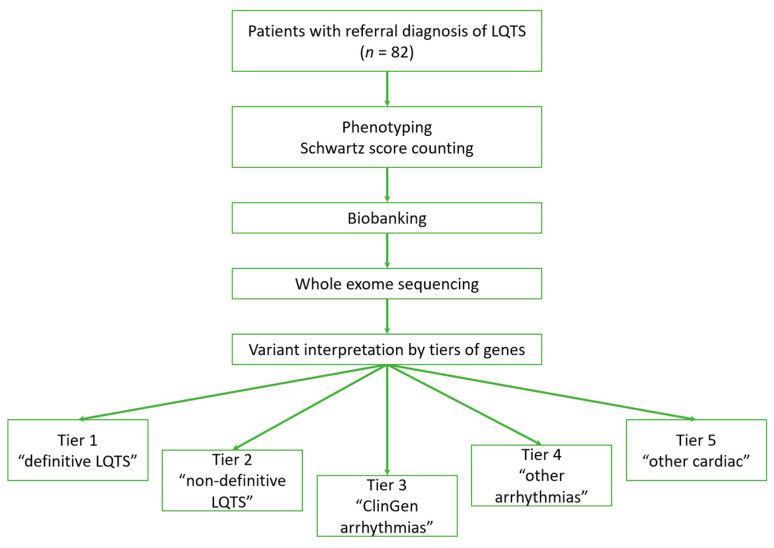
The study workflow.

The graphical summary of the study process is presented in Figure 1.

### 2.1. Characterization of the Cohort

The final cohort consisted of 82 participants from 40 unrelated families. Male-to-female (M:F) ratio in the cohort is 30:52, with a statistically significant predominance of females (63%, *p* = 0.02); the median age is 23 (19; 40) years. Among 40 index patients, the M:F ratio is 13:27 (68% females), and the median age is 21 (19; 30) years.

**Figure 2:**
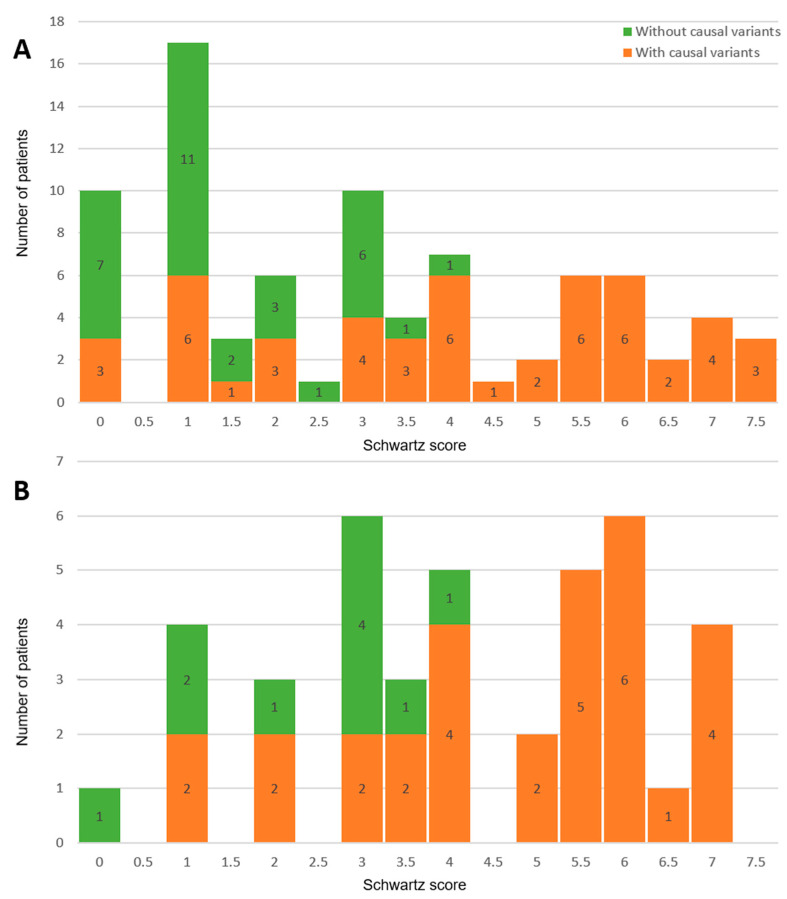
Distribution of Schwartz score and LQTS-causing variants: (**A**) in the whole cohort and (**B**) in index patients.

The median Schwartz score counted across the cohort is 3 (Figure 2A). For index patients, i.e., those directly referred with the diagnosis of congenital LQTS, the median Schwartz score counted is 4 (Figure 2B). It is noteworthy that 14 of them (35%) did not reach the formal Schwartz threshold of 3.5 [5] for the “high probability” of the disease.

Clinical characteristics of the cohort obtained by analysis of the medical history and instrumental data and used for the Schwartz score counting are summarized in Table 1. Index patients had a significantly longer median QTc and a higher incidence of syncopes compared to their relatives. The carriers of causal variant(s) had more severe disease (significantly longer median QTc and higher incidence of syncopes and torsade de pointe episodes) than non-carriers.

### 2.2. Genetic Testing Results

#### 2.2.1. Diagnostic Testing for LQTS-Causing Variants

**Figure 3:**
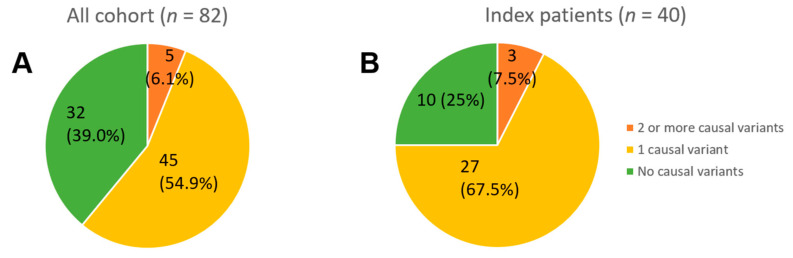
The yield of causal variants for LQTS: (**A**) in the whole cohort and (**B**) in index patients.

The yield of causal variants (in the genes from Tier 1—“definitive LQTS”) was 61% in the whole studied cohort and 75% in index patients. Five patients (6.1%) had two or more causal variants, and three of them were index patients (Figure 3). 

All LQTS-causing variants are concentrated in three major well-known genes (*KCNQ1*, *KCNH2*, *SCN5A*). Of note, the variants in *KCNQ1* and *KCNH2* are highly prevalent (93.1% of all causal variants found). The majority of causal findings are missenses (75.8%). The spectrum of LQTS-causing findings in our patients is presented in Table 2. 

#### 2.2.2. Extended Research Testing

**Figure 4:**
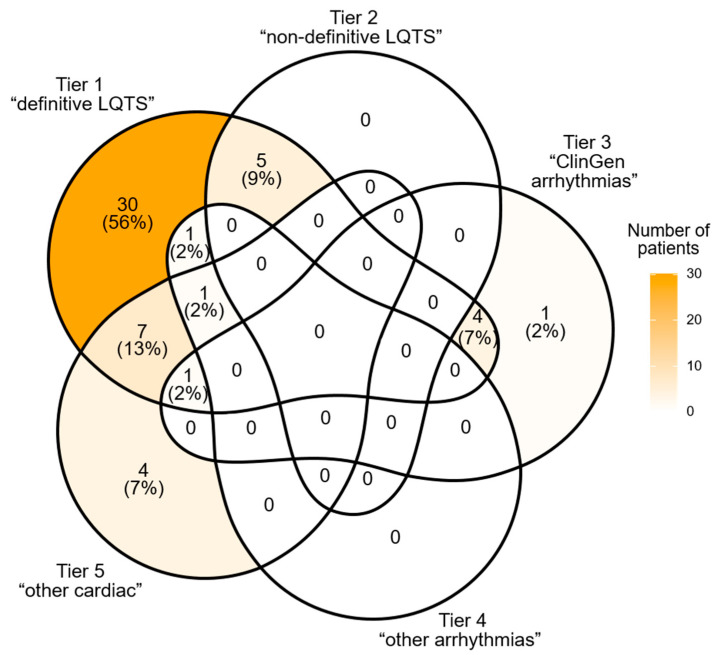
Venn diagram showing the distribution of genetic findings among patients.

At least one variant in any of the 248 studied genes was found in 55 (67%) patients. However, there were only five persons (four of them were index patients) who had a finding in Tiers 3, 4, or 5 (non-LQTS genes) but no findings in Tiers 1 and 2 (Figure 4). One index patient of the aforementioned four harbored the variant in the gene from Tier 5 (*PKP2*) that was interpreted as causal for arrhythmogenic cardiomyopathy (ARVC). The spectrum of findings in Tiers 2–5 (beyond the set of causal genes) is presented in Table 3.

**Figure 5:**
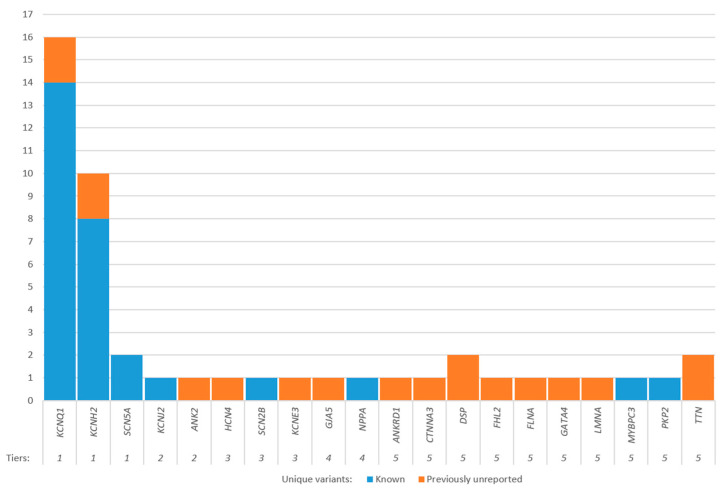
Distribution of unique genetic variants found in this study by genes.

Overall, we observed 48 unique genetic variants in the studied cohort, of which 29 are causal for LQTS, and one is causal for ARVC. Of the total number of variants found, 18 had not been previously described, but only four of these belong to the “definitive LQTS” tier of genes (Figure 5).

Notably, six out of fourteen (42.9%) index patients with a Schwartz score <3.5 were found to harbor an LQTS-causing variant. Moreover, the patient with the ARVC-causing variant mentioned above also had a Schwartz score <3.5; therefore, seven out of fourteen patients with low Schwartz scores received the precise molecular genetic diagnosis after extended genetic testing.

### 2.3. Evaluating the Impact of Additional Rare SNPs

The carriers of variants from Tiers 1 (“definitive LQTS”) and 5 (“other cardiac”) had significantly higher Schwartz scores than non-carriers (Table 4). It is noteworthy that the significance of this difference was substantially more valid for the variants from Tier 1 (*p* value < 0.001) than for Tier 5 (*p* value = 0.022). No statistically significant association was observed for the carriers of variants belonging to Tiers 2, 3, and 4; notably, the number of findings in these tiers was very low.

A multivariate regression analysis with an adjustment for family as a random effect was conducted to evaluate how the severity of LQTS changes per each additional genetic variant from Tiers 1 to 5 (Table 5).

As it can be seen, each finding in the genes of Tier 1 gave 2.4 additional points to the Schwartz score, contributing to a more severe phenotype in the carriers of two or more “definitive” variants. Surprisingly, the carriers of variants belonging to Tier 2 (“non-definitive LQTS”) demonstrated a likely “decrease” in the Schwartz score compared to the carriers of causal variants. In order to understand whether this reflects the real changes in phenotype severity, we retrospectively analyzed medical records and found that all variants from “non-definitive LQTS” genes were harbored by the members of the only family who also had causal variants in *KCNQ1*. Thus, the result for Tier 2 variants was considered biased, and these variants were excluded from the subsequent analysis. 

Moreover, considering the tendency to statistical significance (*p* value = 0.065) of the Schwartz score increase per each finding in Tier 5 (“other cardiac” genes) and given the small number of findings in the genes from Tier 4 (“other arrhythmias”, having a rather indirect relation to the LQTS pathogenesis, as well as the genes from Tier 5), Tiers 4 and 5 were merged into one category of “non-ClinGen cardiac genes” for the next step of the analysis. The results of these modified calculations are presented in Table 6.

The presence of rare SNPs in non-ClinGen cardiac genes resulted in a significant increase in the Schwartz score of 0.94 per finding (CI: 0.06; 1.82; *p* value = 0.04). Statistical significance of this observation was reached after merging Tiers 4 and 5 due to the subsequent increase in the number of variants in the analyzed category. Thus, in our cohort, the patients harboring rare cardiac genetic variants in addition to the definitive LQTS-causing variants showed more phenotypic severity than the carriers of LQTS-causing variants only.

## 3. Discussion

Over the last decades, the strategy of DNA diagnosis for LQTS and other cardiac channelopathies has evolved along with the developing technologies and standards of genetic testing: from targeted consecutive sequencing of single genes to simultaneous investigation of tens of genes by next-generation sequencing [47,48], then to an evidence-based approach and critical reassessment of accumulated data in order to narrow and prioritize the list of relevant genes [49,50]. Through these improvements, the positive yield of genetic testing for LQTS has reached 75–80% [51,52]. In our study, causal findings in either *KCNQ1*, *KCNH2*, or *SCN5A* were detected in 75% of the index patients, which corresponds to the reported worldwide estimates [51], and the vast majority (25/29, 86.2%) of these findings are established LQTS-causing variants, previously described by reputable sources. It is worth mentioning that our index patients were subjected to genetic testing upon referral by their cardiologist to the Expert Arrhythmology Center, without rigorous selection and restrictions on the Schwartz score. Thus, a high positive yield of DNA diagnosis is maintained when the test is prescribed based on clinical suspicion made by an expert cardiologist.

No clinically relevant variants in *CALM1-3* and *TRDN* were found in our cohort. This is not surprising per se, as these genes have been reported to be causative with “definitive” evidence only in the specific rare childhood-onset form of LQTS with cardiac conduction impairment [50]. Nevertheless, the ClinGen curation placed *CALM1-3* and *TRDN* at the same tier of validity as the three major LQTS genes, at the same time stating that their role in “usual” adult-onset LQTS is yet to be determined. Our work shows no impact of these genes in the routine clinical practice of our Arrhythmology Center, although our sample size is limited. Thus, in most cases, the analysis of three major genes is sufficient to provide the answer and to issue the clinically actionable report on DNA diagnosis. However, in the remaining cases, wider testing may be useful.

### 3.1. Patients with “Low or Intermediate” Schwartz Score

A significant proportion of our cohort (57% of the total cohort and 35% of index patients) had Schwartz scores of 3 and lower, thus having a low or intermediate probability of LQTS [5]. As per current recommendations [3], genetic testing is not mandatory for these patients, and only definitive LQTS genes should be analyzed. In recent years, being concerned about the growing overdiagnosis of LQTS and unnecessary medical interventions [53], reputable clinical institutions tend to be even more strict, considering individuals with a Schwartz score <1 as the average population with no need for genetic testing [54]. However, worldwide clinical practice shows quite a few examples of genetically confirmed LQTS with low Schwartz scores [55,56,57]. In our study, 17 patients with a Schwartz score <3.5 from 10 unrelated families were found to carry a pathogenic or likely pathogenic variant in one of three major LQTS genes. Moreover, among index patients with a Schwartz score <3.5, six (42.9%) were carriers of an LQTS-causing variant, and one had a pathogenic frameshift variant in *PKP2*, the major ARVC gene [43]. Since the *PKP2* gene is not included in the recommended scope of DNA diagnosis for those with “low or intermediate” Schwartz probability of LQTS, we may say that, in our case, the extended genetic testing has increased the rate of accurate molecular diagnosis from 42.9 to 50% in this group of patients referred as having LQTS. Of note, being aware of the risks associated with erroneous diagnosis of LQTS, we emphasize that genetic testing in the patients with a Schwartz score <3.5 requires especially meticulous variant interpretation and must not result in reporting of variants of uncertain significance (VUSs), even in well-established LQTS genes.

### 3.2. Impact of Variants from Different Tiers of Genes on LQTS Severity

In our study, the LQTS patients with causal genetic findings (genotype-positive patients) carrying additional rare variants in heart-related genes had significantly higher Schwartz scores than the genotype-positive patients without any additional findings. In general, the presence of relatively rare modifier variants is one of the most obvious ways to explain the clinical heterogeneity of LQTS [58]. However, only a limited number of SNPs with established impact on the phenotype have been described to date [59], mainly in the well-studied genes of ion channels. There are also a number of case reports reviewing additional rare findings in LQTS patients [60,61]. At the same time, the search for possible modifiers is usually limited to the genes with an already known relationship to LQTS, so a number of genes may escape the researchers’ attention since their connection to arrhythmias is yet undiscovered [62,63]. Given the prominent clinical overlap between inherited arrhythmias and cardiomyopathies and the likely common genetic substrate of these two large groups of inborn cardiac conditions [64], we did not limit our search for rare variants in LQTS patients to the known proarrhythmic genes, including the genes of structural and regulatory myocardial proteins to the Tier 5 (“other cardiac” genes) of our research gene list.

Our results not only underscore the known fact that carriers of pathogenic genetic variants tend to have a more severe phenotype [65] but also show that genotype-positive LQTS patients harboring additional rare variants in genes related to cardiac morphology and regulation tend to have higher Schwartz scores. Until now, the possible impact of rare variants in these genes has been discussed in the context of genotype-negative LQTS patients, i.e., in the absence of established causal variants [66,67]. In the current clinical setting, such findings have no prognostic and therapeutic implications and must not be included in any reports to the patients and/or their treating cardiologists. In the research setting, however, these rare SNPs in cardiac genes, observed in co-occurrence with known disease-causing variants, may be considered potentially functional variants whose impact on the phenotype remains to be evaluated. The molecular mechanisms underlying this possible role cannot be discussed until these observations are reproduced or functional studies are performed. However, we found notable that the substantial proportion of our findings in Tiers 4 and 5 affects proteins of cell contacts in cardiomyocytes, such as desmosome components (*DSP*, *PKP2*), gap junction components (*GJA5*), or adhesion proteins (*CTNNA3*) (see Table 3). It is known that damaging variants in desmosome proteins provide an arrhythmogenic substrate [68,69] in several ways, including impairment of sodium current [70]. We suppose that the arrhythmogenesis from impaired ion channels may be aggravated by the substrate of compromised cell–cell contacts, thus worsening the disease course in such patients. We suggest that in terms of a search for the causes of LQTS phenotypic heterogeneity, sharing of VUSs found in genotype-positive and genotype-negative patients in research publications may be potentially helpful for future research, as gene–disease associations may be established through the accumulation of the genetic data across researchers.

### 3.3. Limitations of This Study

The overall statistical power of this study is limited due to the small size of the cohort.

Unfortunately, we could not collect comprehensive medical data from some patients, which may affect our estimates of the Schwartz score.

We did not analyze the associations between variant(s) occurrence and distinct cardiac events (syncope, torsade de pointe episodes, etc.) because, given the small sample size, we considered it more appropriate to use the Schwartz score—a comprehensive value reflecting all aspects of disease severity—to ensure obtaining the quality statistical results.

The workflow for finding and interpreting genetic variants of interest used in this study was tailored to rare (MAF <3%) variants; thus, the possible impact of some reported modifiers with a high MAF [59] was not considered.

## 4. Materials and Methods

### 4.1. Cohort Selection and Clinical Examination

The study cohort was retrospectively recruited from patients consulted in an outpatient clinic or admitted to the Expert Arrhythmology Center of the National Medical Research Center for Therapy and Preventive Medicine (Moscow, Russia) with a referral diagnosis of congenital LQTS from 2018 to 2021. All index patients had their disease and family history taken and underwent 12-lead supine electrocardiography (ECG), blood samples biobanking, genetic testing, and cardiogenetic counseling. Additional medical test results (including Holter ECG, exercise stress test, etc.) were requested if available. First- and second-degree relatives were invited if available and examined in the same way as the index patients. The study was performed in accordance with the Declaration of Helsinki and approved by the Ethics Committee of the National Medical Research Center for Therapy and Preventive Medicine. All participants (or their legal representatives, if appropriate) gave their written informed consent to the study.

The QT interval assessment was performed using a 12-lead ECG recorded under resting conditions. The QT interval was measured manually in the lead V5 or V6 from the beginning of the QRS complex to the end of the T wave defined by the tangent method. Correction for rate was performed using the Bazett formula; in cases of heart rate less than 60 beats per minute and over 100 beats per minute, the Framingham method was used.

The LQTS score was determined using modified Schwartz LQTS diagnostic criteria [5].

### 4.2. Sequencing and Bioinformatic Analysis

DNA was isolated from venous blood samples using the QIAamp DNA Blood Mini Kit (Qiagen, Hilden, Germany) with the following quality control on a Qubit 4.0 fluorimeter (Thermo Fisher Scientific, Waltham, MA, USA). Whole exome sequencing was carried out with IDT-Illumina TruSeq DNA exome libraries on NextSeq 550 (Illumina, San Diego, CA, USA) in accordance with the manufacturer’s protocols. Sequencing reads were aligned to the reference genome (GRCh38) using bwa-mem [71], and nucleotide variants were called with GATK 4.2 HaplotypeCaller [72] and annotated using Ensembl Variant Effect Predictor [73]. All genes connected to LQTS according to the current consensus recommendations for DNA diagnosis of channelopathies [3] were included in the analysis. In terms of expanded testing and deeper investigation, we also included the genes connected with other channelopathies, congenital heart disease, and primary cardiomyopathies, i.e., all genes that may contribute to arrhythmogenic substrates, to a total of 248 genes analyzed (Supplementary Table S1). The validation of candidate variants was performed by Sanger sequencing using the ABI PRISM BigDye Terminator Reagent Kit v. 3.1 (Thermo Fisher Scientific, Waltham, MA, USA) on the Applied Biosystems 3500 Genetic Analyzer (Thermo Fisher Scientific, Waltham, MA, USA).

### 4.3. Algorithm of Analysis of Genetics Findings

For clinical interpretation, we selected nucleotide variants with a minor allele frequency (MAF) less than 3% across populations in gnomAD v3.1.2 [74], i.e., all variants that do not fit the standalone benign criteria as per national consensus recommendations for variant interpretation [75]. The pathogenicity assessment was performed in accordance with the standard algorithm recommended by the ACMG/AMP consensus guidelines for the interpretation of sequence variants [37]. For the variants in LQTS-causing genes, we used the disease-specific implementations of ACMG/AMP guidelines provided by the field experts [38,50].

To evaluate how the variants in different genes contribute to the LQTS phenotype, we divided all investigated genes into five tiers based on the proximity of the gene products to the pathogenetic pathway of QT prolongation. Tier 1 (“definitive LQTS”) consists of seven genes with “definitive” and “strong” gene–disease correlations with LQTS according to ClinGen curation. Tier 2 (“non-definitive LQTS”) contains 10 genes with lower (“moderate”, “limited”, and “disputed”) ClinGen levels of gene–disease validity for LQTS. Tier 3 (“ClinGen arrhythmias”) includes 20 genes assigned in ClinGen to any other primary arrhythmias with any level of gene–disease validity. Tier 4 (“other arrhythmias”) consists of 60 genes associated with heart rhythm disorders according to the literature or reputable databases, but not respectively curated by ClinGen. The remaining 151 genes related to other cardiac diseases and heart morphogenesis make up Tier 5 (“other cardiac”). Variants in the genes assigned to Tier 1 were considered causal in accordance with current recommendations for genetic testing. The full list of genes included in all five tiers can be found in Supplementary Table S1. The Schwartz score was used as the measure of LQTS phenotype severity.

### 4.4. Statistical Analysis

Statistical analysis was performed using R 4.2. Continuous parameters are presented as mean and standard deviation (M ± SD) or median and interquartile range (Me (Q25; Q75)). Qualitative indicator variables are described by frequencies. The Mann–Whitney U-test was used to compare distributions of continuous variables, and Fisher’s two-tailed exact test was used to compare categorical variables. The association between genetic variants and Schwartz scale scores was assessed using multivariate linear regression with random effects. The intercept was treated as a random effect in each family considered. For each regression, the number of variants in the corresponding gene tier was included as a predictor. No adjustment was made for other covariates. No adjustment for multiple comparisons was performed. The significance level was assumed to be 0.05.

## 5. Conclusions

In general, our work confirms the known genotype–phenotype correlations in LQTS and shows the robustness of the DNA diagnosis framework focusing on the main definitive genes. At the same time, it demonstrates that disregarding genetic testing in index patients with a low or intermediate probability of diagnosis based on the Schwartz score may be unreasonable. Our results indicate the need for further studies in order to establish the target group for genetic testing among individuals with the borderline LQTS phenotype.

Moreover, our study shows that additional rare variants in the genes of structural and regulatory myocardial proteins may affect the clinical severity of LQTS in the carriers of causal genetic variants in definitive genes, although these observations need to be extended and reproduced before any conclusions can be drawn about their potential clinical significance.

## Figures and Tables

**Table 1:** Clinical characteristics of the cohort.

	Total, *n* = 82	Index, *n* = 40	Relatives, *n* = 42	Patients with Causal Variants, *n* = 50	Patients Without Causal Variants, *n* = 32
Median age, years	23 (19; 40)	21 (19; 30)	33 (16; 44)	21 (19; 34)	32 (15; 41)
Number of patients with ECG, *n*	72	40	32	46	26
Median QTc, ms	472 (433; 501)	482 (457; 513)	439 (411; 478) *	488 (465; 520)	436 (403; 460) **
QTc ≥480 ms during 4th minute of recovery from an exercise test, %	86.7 (13/15)	100 (9/9)	66.7 (4/6)	100 (9/9)	66.7 (4/6)
Torsade de pointe arrhythmia, %	15.6 (12/77)	25.6 (10/39)	5.3 (2/38)	26.1 (12/46)	0 ** (0/31)
T wave alternans, %	4.2 (3/72)	5 (2/40)	3.1 (1/32)	6.5 (3/46)	0 (0/26)
Notched T wave in three leads, %	12.5 (9/72)	12.5 (5/40)	12.5 (4/32)	19.6 (9/46)	0 ** (0/26)
Resting heart rate < second percentile for age, %	28.8 (15/52)	28.6 (8/28)	29.2 (7/24)	36.4 (12/33)	15.8 ** (3/19)
Syncope, %	45.5 (35/77)	69.2 (27/39)	21.1 * (8/38)	60.9 (28/46)	22.6 ** (7/31)
Stress-induced syncope, %	19.5 (15/77)	30.8 (12/39)	7.9 * (3/38)	28.3 (13/46)	6.5 ** (2/31)
Congenital deafness, %	0 (0/82)	0 (0/40)	0 (0/42)	0 (0/50)	0 (0/32)
Family history, %	63.4 (52/82)	47.5 (19/40)	78.6 (33/42)	70 (35/50)	53.1 (17/32)

* *p* < 0.05 for difference between index patients and relatives. ** *p* < 0.05 for difference between the patients with and without causal variants.

**Table 2:** LQTS-causing genetic variants observed in the studied cohort.

Variant No.	hg38	Genotype	Gene	Coding	Protein	Variant Type	Protein Region	Class	No. of Patients	No. of Families	First Reported
1	chr11:2528019	G>A	*KCNQ1*	c.477+1G>A		Splice	Transmembrane/Linker/Pore	P	3	2	[18]
2	chr11:2778015	G>A	*KCNQ1*	c.1772G>A	p.Arg591His	Missense	C-terminus (highly conserved)	P	4	1	[19]
3	chr11:2572922	A>G	*KCNQ1*	c.857A>G	p.Asp286Gly	Missense	Transmembrane/Linker/Pore	LP	3	1	Present study
4	chr11:2570671	G>T	*KCNQ1*	c.521G>T	p.Arg174Leu	Missense	Transmembrane/Linker/Pore	LP	2	1	[20]
5	chr11:2445419	G>T	*KCNQ1*	c.321G>T	p.Gln107His	Missense	N-terminus	LP	2	1	[21]
6	chr11:2570740	C>T	*KCNQ1*	c.590C>T	p.Pro197Leu	Missense	Transmembrane/Linker/Pore	VUS	1	1	[22]
7	chr11:2583535	C>T	*KCNQ1*	c.1022C>T	p.Ala341Val	Missense	Transmembrane/Linker/Pore	P	1	1	[23]
8	chr11:2570719	G>A	*KCNQ1*	c.569G>A	p.Arg190Gln	Missense	Transmembrane/Linker/Pore	P	1	1	[23]
9	chr11:2583457	A>G	*KCNQ1*	c.944A>G	p.Tyr315Cys	Missense	Transmembrane/Linker/Pore	P	1	1	[24]
10	chr11:2776997	C>A	*KCNQ1*	c.1697C>A	p.Ser566Tyr	Missense	C-terminus (highly conserved)	P	1	1	[25]
11	chr11:2778009	G>A	*KCNQ1*	c.1766G>A	p.Gly589Asp	Missense	C-terminus (highly conserved)	P	2	2	[26]
12	chr11:2572104	C>T	*KCNQ1*	c.775C>T	p.Arg259Cys	Missense	Transmembrane/Linker/Pore	P	1	1	[27]
13	chr11:2585275	C>T	*KCNQ1*	c.1096C>T	p.Arg366Trp	Missense	C-terminus (highly conserved)	P	1	1	[24]
14	chr11:2662025-2664532	deletion of 2507 bp	*KCNQ1*	c.1459_1514+2451delinsCTGAGGAAACAGGGCAC		Structural variant	C-terminus	LP	1	1	Present study
15	chr11:2583434	G>C	*KCNQ1*	c.922-1G>C		Splice	Transmembrane/Linker/Pore	P	1	1	[28]
16	chr11:2570713	G>A	*KCNQ1*	c.563G>A	p.Trp188Ter	Nonsense	Transmembrane/Linker/Pore	P	1	1	[29]
17	chr11:2583453	G>A	*KCNQ1*	c.940G>A	p.Gly314Ser	Missense	Transmembrane/Linker/Pore	P	1	1	[30]
18	chr7:150977846	T>C	*KCNH2*	c.68A>G	p.Glu23Gly	Missense	N-terminus LQTS cluster	LP	1	1	Present study
19	chr7:150952763	T>C	*KCNH2*	c.1219A>G	p.Lys407Glu	Missense	Transmembrane/Linker/Pore	LP	1	1	[31]
20	chr7:150952691	A>G	*KCNH2*	c.1291T>C	p.Phe431Leu	Missense	Transmembrane/Linker/Pore	LP	3	1	[29]
21	chr7:150948449	TCCGTGCG>T	*KCNH2*	c.2680_2686del	p.Arg894Thrfs78	Frameshift	C-terminus	LP	2	1	Present study
22	chr7:150958449	G>A	*KCNH2*	c.526C>T	p.Arg176Trp	Missense	N-terminus	LP	7	5	[26]
23	chr7:150952481	C>A	*KCNH2*	c.1501G>T	p.Asp501Tyr	Missense	Transmembrane/Linker/Pore	P	3	1	[32]
24	chr7:150950421	CTGGGGGCAGGG>C	*KCNH2*	c.2146-12_2146-2del		Splice	C-terminus	LP	1	1	[33]
25	chr7:150951555	G>A	*KCNH2*	c.1838C>T	p.Thr613Met	Missense	Transmembrane/Linker/Pore	P	1	1	[34]
26	chr7:150948455	C>CGCCTG	*KCNH2*	c.2676_2680dup	p.Arg894ProfsTer82	Frameshift	C-terminus	P	1	1	[29]
27	chr7:150974924	C>T	*KCNH2*	c.94G>A	p.Ala32Thr	Missense	N-terminus LQTS cluster	LP	1	1	[29]
28	chr3:38551022	C>T	*SCN5A*	c.5350G>A	p.Glu1784Lys	Missense	C-terminus	P	7	2	[35]
29	chr3:38551442	G>A	*SCN5A*	c.4930C>T	p.Arg1644Cys	Missense	Transmembrane	LP	1	1	[36]

Classes of pathogenicity are defined as per ACMG/AMP guidelines [37]: LP, likely pathogenic; P, pathogenic; and VUS, variant of uncertain significance. Protein regions are listed according to the disease-specific interpretation criteria provided by Walsh et al. [38].

**Table 3:** Spectrum of genetic variants in additional (non-LQTS-causing) genes observed in the studied cohort.

Tier of Genes	hg38	Genotype	Gene	Coding	Protein	Variant Type	Gene Product Function *	Class	No. of Patients	No. of Families	First Reported	Co-Occurring Causal Variant No. **
“non-definitive LQTS” (2)	chr4:113369675	A>G	*ANK2*	c.11480A>G	p.Gln3827Arg	Missense	Cytoskeletal anchoring in cardiomyocytes	VUS	3	1	Present study	2; 3; 2 + 3
“non-definitive LQTS” (2)	chr17:70175316	G>A	*KCNJ2*	c.277G>A	p.Val93Ile	Missense	Cardiac voltage-gated potassium channel	VUS	3	1	[39]	2; 2 + 3 (2)
“Clingen arrhythmias” (3)	chr15:73325009	C>T	*HCN4*	c.1924G>A	p.Val642Met	Missense	Cardiac cyclic nucleotide-gated potassium channel	VUS	3	1	Present study	28 (2)
“Clingen arrhythmias” (3)	chr11:74457538	G>A	*KCNE3*	c.26C>T	p.Thr9Ile	Missense	Cardiac voltage-gated potassium channel	VUS	1	1	Present study	22
“Clingen arrhythmias” (3)	chr11:118168740	G>A	*SCN2B*	c.82C>T	p.Arg28Trp	Missense	cardiac voltage-gated sodium channel	VUS	2	1	[40]	21 (2)
“other arrhythmias” (4)	chr1:147758395	G>T	*GJA5*	c.844C>A	p.Pro282Thr	Missense	Gap junction component	VUS	1	1	Present study	13
“other arrhythmias” (4)	chr1:11847213	G>A	*NPPA*	c.350C>T	p.Ala117Val	Missense	Peptide hormone involved in cardiac remodeling and apoptosis regulation	VUS	1	1	[41]	22 + 27
“other cardiac” (5)	chr10:90920258	CTAAAG>C	*ANKRD1*	c.113_117del	p.Thr38ArgfsTer43	Frameshift	Transcription factor (TF) involved in sarcomere organization	VUS	1	1	Present study	21
“other cardiac” (5)	chr10:66379166	A>T	*CTNNA3*	c.1718T>A	p.Phe573Tyr	Missense	Cell–cell adhesion in muscle cells	VUS	1	1	Present study	28
“other cardiac” (5)	chr6:7568519	C>T	*DSP*	c.1349C>T	p.Pro450Leu	Missense	Desmosome component	VUS	2	1	Present study	4 (2)
“other cardiac” (5)	chr6:7584911	G>A	*DSP*	c.7649G>A	p.Gly2550Asp	Missense	Desmosome component	VUS	1	1	Present study	13
“other cardiac” (5)	chr2:105363302	C>T	*FHL2*	c.671G>A	p.Cys224Tyr	Missense	TF expressed in heart tissue	VUS	1	1	Present study	29
“other cardiac” (5)	chrX:154357257	C>T	*FLNA*	c.4963G>A	p.Gly1655Arg	Missense	Cytoskeleton component	VUS	1	1	Present study	None ***
“other cardiac” (5)	chr8:11750121	G>A	*GATA4*	c.797G>A	p.Arg266His	Missense	TF involved in myocardial differentiation	VUS	1	1	Present study	None ***
“other cardiac” (5)	chr1:156115222	C>G	*LMNA*	c.304C>G	p.Leu102Val	Missense	Cytoskeleton component	VUS	1	1	Present study	None
“other cardiac” (5)	chr11:47339742	A>G	*MYBPC3*	c.1976T>C	p.Ile659Thr	Missense	Sarcomere protein	VUS	1	1	[42]	7
“other cardiac” (5)	chr12:32878524	TA>T	*PKP2*	c.355del	p.Tyr119MetfsTer23	Frameshift	Desmosome component	P	2	1	[43]	None (2)
“other cardiac” (5)	chr2:178615372	T>C	*TTN*	c.48573A>G	p.Ile16191Met	Missense	Major sarcomere protein	VUS	1	1	Present study	22
“other cardiac” (5)	chr2:178741501	GA>G	*TTN*	c.11731del	p.Ser3911ProfsTer34	Frameshift	Major sarcomere protein	LP	1	1	Present study	10

* Gene function was curated from NCBI Genes [44], UniProtKB/Swiss-Prot [45], and the Human Protein Atlas [46] databases. ** The LQTS-causing variant(s) are indicated by the numbers assigned in the “Variant No.” (left-most) column of Table 2. In cases where the same combination of causal and additional variants is harbored by more than one patient, the number of patients is indicated in parentheses. *** Two variants harbored by the same patient.

**Table 4:** Comparison of LQTS severity between carriers and non-carriers in dependence of the tier of variants.

Tier	Schwartz Score in Carriers, Points	Schwartz Score in Non-Carriers, Points	*р* Value
“definitive LQTS” (1)	4.14 ± 2.25	1.50 ± 1.19	<0.001
“non-definitive LQTS” (2)	2.40 ± 2.86	3.16 ± 2.28	0.44
“ClinGen arrhythmias” (3)	4.17 ± 2.36	3.03 ± 2.29	0.23
“other arrhythmias” (4)	6.00 ± 0.00	3.04 ± 2.28	0.08
“other cardiac” (5)	4.38 ± 1.80	2.87 ± 2.32	0.022

**Table 5:** Impact of rare variants in cardiac genes on the Schwartz score (per finding in each tier).

Tier	Mean Increase in the Schwartz Score per Variant (CI) ^1^	*p* Value
“definitive LQTS” (1)	2.43 (1.75 to 3.10)	<0.001
“non-definitive LQTS” (2)	−2.34 (−4.14 to −0.54)	0.016
“ClinGen arrhythmias” (3)	−0.10 (−1.64 to 1.44)	0.90
“other arrhythmias” (4)	0.37 (−2.27 to 3.01)	0.77
“other cardiac” (5)	0.91 (−0.04 to 1.86)	0.065

^1^ CI—confidence interval.

**Table 6:** Impact of rare variants on the Schwartz score (modified calculations for ClinGen-associated proarrhythmic genes and non-ClinGen cardiac genes).

Tier	Mean Increase in the Schwartz Score per Variant (CI) ^1^	*p* Value
1	2.19 (1.53 to 2.85)	<0.001
3	0.01 (−1.57 to 1.59)	0.99
4 + 5	0.94 (0.06 to 1.82)	0.04

^1^ CI—confidence interval.

## Data Availability

The data presented in this study are available upon request from the corresponding author.

## Supplementary Materials

The following supporting information can be downloaded at: https://www.mdpi.com/article/10.3390/ijms252211976/s1.
